# Auditory Processing of Speech and Tones in Children With Tuberous Sclerosis Complex

**DOI:** 10.3389/fnint.2020.00014

**Published:** 2020-04-09

**Authors:** Amanda M. O’Brien, Laurie Bayet, Katherine Riley, Charles A. Nelson, Mustafa Sahin, Meera E. Modi

**Affiliations:** ^1^Program in Speech and Hearing Bioscience and Technology, Division of Medical Sciences, Harvard University, Cambridge, MA, United States; ^2^Department of Psychology, American University, Washington, DC, United States; ^3^Laboratories of Cognitive Neuroscience, Division of Developmental Medicine, Boston Children’s Hospital, Boston, MA, United States; ^4^Harvard Graduate School of Education, Harvard University, Cambridge, MA, United States; ^5^Translational Neuroscience Center, Boston Children’s Hospital, Boston, MA, United States; ^6^Department of Neurology, Harvard Medical School, Boston, MA, United States

**Keywords:** Tuberous Sclerosis Complex, autism spectrum disorder, auditory evoked potential, MVPA, mismatch negativity

## Abstract

Individuals with Tuberous Sclerosis Complex (TSC) have atypical white matter integrity and neural connectivity in the brain, including language pathways. To explore functional activity associated with auditory and language processing in individuals with TSC, we used electroencephalography (EEG) to examine basic auditory correlates of detection (P1, N2, N4) and discrimination (mismatch negativity, MMN) of speech and non-speech stimuli for children with TSC and age- and sex-matched typically developing (TD) children. Children with TSC (TSC group) and without TSC (typically developing, TD group) participated in an auditory MMN paradigm containing two blocks of vowels (/a/and/u/) and two blocks of tones (800 Hz and 400 Hz). Continuous EEG data were collected. Multivariate pattern analysis (MVPA) was used to explore functional specificity of neural auditory processing. Speech-specific P1, N2, and N4 waveform components of the auditory evoked potential (AEP) were compared, and the mismatch response was calculated for both speech and tones. MVPA showed that the TD group, but not the TSC group, demonstrated above-chance pairwise decoding between speech and tones. The AEP component analysis suggested that while the TD group had an increased P1 amplitude in response to vowels compared to tones, the TSC group did not show this enhanced response to vowels. Additionally, the TD group had a greater N2 amplitude in response to vowels, but not tones, compared to the TSC group. The TSC group also demonstrated a longer N4 latency to vowels compared to tones, which was not seen in the TD group. No group differences were observed in the MMN response. In this study we identified features of the auditory response to speech sounds, but not acoustically matched tones, which differentiate children with TSC from TD children.

## Introduction

Tuberous Sclerosis Complex is a genetic syndrome caused by a mutation in either the *TSC1* or *TSC2* gene. TSC is characterized by the formation of lesions on multiple organs including the brain, skin, kidneys, and lungs. Concurrent with TSC, approximately 50% of individuals are co-diagnosed with intellectual disabilities and 20–60% are co-diagnosed with ASD (Ehninger et al., 2009; Mcdonald et al., 2017), which contribute to pervasive deficits in language acquisition and development (Prather and De Vries, 2004).

Underlying these neurodevelopmental impairments, patients with TSC present with abnormalities in white matter microstructure (Peters et al., 2013), particularly within language pathways (Lewis et al., 2013). Molecular evidence suggests that the reduction in white matter in TSC is due to decreased myelination, altered axonal arborization, and synaptic formation (Tavazoie et al., 2005; Meikle et al., 2007; Choi et al., 2008; Ercan et al., 2017). While it is hypothesized that such structural differences in the brain lead to auditory and language deficits in TSC, the neural response to basic auditory and speech stimuli is not well characterized. Electroencephalography (EEG) can be used to determine if there are functional alterations in addition to structural deviations in patients with TSC. EEG is ideally suited for assessing functional activity with high temporal resolution in young and neurodevelopmentally delayed populations, as it is non-invasive and does not require active participation (Jeste et al., 2015). Further, the high temporal resolution of EEG is ideal for a time-locked exploration of early auditory processing.

In this study, we explored the neural processing of tones and speech sounds in children with TSC. The neural responses to auditory stimuli in a mismatch negativity (MMN) paradigm was compared in children with and without TSC using auditory evoked response potentials (AEP), time-resolved multivariate pattern analysis (MVPA), and MMN analysis. MVPA considers complete neural activation patterns at each individual time point, rather than focusing on one specific region and time point of interest (Cauchoix et al., 2014; Grootswagers et al., 2017; Holdgraf et al., 2017; Bayet et al., 2018). Thus, MVPA allows for the exploration of potential compensatory mechanisms of processing (i.e., unique localizations and patterns) that may be established in clinical populations due to structural aberrations. To our knowledge, this study is the first to utilize MVPA for speech sound processing in a pediatric population.

The AEP, elicited by an auditory stimulus and collected using EEG, is a traditional measure of basic auditory detection that is well conserved in typically developing populations (Picton, 2011; O’connor, 2012). Deviations from the stereotyped response, therefore, serve as an apt measure of differences in functional auditory detection and may serve as biomarkers of functional impairment in the disorder. Mismatch negativity (MMN) is a second order measure of auditory processing that represents a neural discrimination response induced by an unexpected stimulus (Naatanen et al., 2007, 2012). The MMN reflects learning and habituation while not requiring overt behavioral responses, and is thus an appropriate measure for use with clinical populations (Naatanen et al., 2012).

Early efforts suggest AEP may reflect neural disruptions in TSC. Parallel to the white matter abnormalities seen in neuroimaging in individuals with TSC and ASD, co-diagnosis is also associated with an increased latency in the N1 component of the AEP and a reduction in the MMN response to tones relative to those with TSC alone (Seri et al., 1999). The correlation between neurodevelopmental, neuroimaging and electrophysiological phenotypes in TSC empowers the use of EEG for biomarker detection. Based on the specificity of white matter abnormalities to language pathways and the prevalence of language impairment in children with TSC, we predict that MVPA and AEP analyses of the neural responses to speech and tone stimuli will reveal (1) decreased accuracy of decoding between speech and tones in the TSC population compared to typically developing children, (2) typical early sensory responses but disrupted later cognitive responses, and (3) reduced MMN response to vowel changes, but not tone changes, compared to typically developing children.

## Materials and Methods

### Participants

Eleven children with a clinical diagnosis of TSC between the ages of 4 and 14, and age- and sex- matched typically developing (TD) children (mean age: 9.28) were recruited from the multidisciplinary Tuberous Sclerosis Program of the Department of Neurology at Boston Children’s Hospital. Medical history for both groups including auditory deficits, visual deficits, neurological conditions and current pharmacological treatments were collected through parent questionnaire.

**FIGURE 1:**
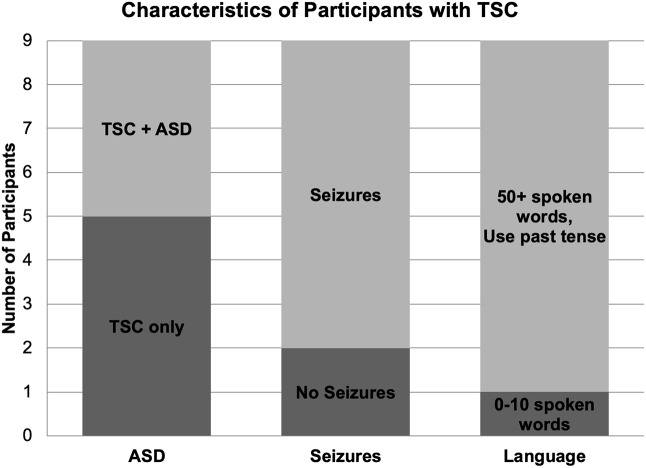
Diagnostic, medical, and language characteristics of participants with TSC. Four of the nine participants with TSC were co-diagnosed with ASD. Seven children with TSC experienced seizures, while two children did not. Per parent report, one participant with TSC used 0–10 spoken words while the other eight children used over 50 spoken words and the past tense.

Nine children (mean age: 9.22; range 9.90, 4 boys) with a diagnosis of TSC were included in the study (Table 1). Two of the eleven recruited children with TSC were excluded due to seizure activity during the test session and excessive movement artifact. Seven participants reported a history of seizures, and five participants were being treated with seizure medication during the study. Four participants with TSC had a clinical co-diagnosis of ASD based on parent report. One participant with TSC was exposed to Spanish as a first language and English as a second language; however, because the speech sound stimuli used in this study are present in both Spanish and English, this participant was not excluded from the study. All other participants in this group were monolingual English speakers (Figure 1).

**TABLE 1:** Demographic and clinical data for participants.

	**Control**	**TSC**
Age	9.24 (4.09–14.36)	9.22 (4.64–14.55)
Sex	5/9 female	5/9 female
Race	9/9 white	8/9 white
Clinical diagnosis	None	5/9 TSC 4/9 TSC + ASD
Hearing/vision	0/9 Hearing Dx 2/9 Vision Dx	0/9 Hearing Dx 3/9 Vision Dx
Language	9/9 L1: English	8/9 L1: English
Speech therapy	0/9	9/9

Nine TD children who were age (± 6 months) and sex-matched to the TSC group (mean age: 9.28; range: 10.26; 4 males) participated in the control group. Per parent report, children in the control group had no history of neurological abnormalities or traumas, birth-related complications, developmental delays, uncorrected vision difficulties, nor immediate family history of neurodevelopmental disorders. Reports were not confirmed with medical records. One participant in the control group had simultaneous language exposure to English and German. Two participants reported English as a first language with some exposure to a second language (Italian, American Sign Language). All other participants in the control group were monolingual English speakers. No participant in either group presented with hearing abnormalities or uncorrected vision difficulties.

The Institutional Review Board at Boston Children’s Hospital approved this study (P00023954). Informed written consent was obtained from the parents of each participant, and from the participants as appropriate.

### Stimuli

The stimuli included two vowel sounds (/a/and/u/) and two non-speech acoustically matched tones (800 Hz and 400 Hz, respectively). A female speaker of American English recorded the vowel sounds using PRATT computer software. Each non-speech tone complement was synthesized with PRATT computer software to be within one standard deviation of the first two formants (F1, F2) of the average female formants and the corresponding recorded speech sounds (Sandoval and Utianski, 2015). Stimuli were each 300 ms in length, with a 0.05 ms on-ramp and off-ramp. Files of the tone and vowel stimuli are included as Supplementary Material.

### Stimuli Presentation

The stimuli were presented in a within-category MMN paradigm (deviant stimuli 15%, never in succession). Participants listened to four stimuli blocks; each block contained 360 pseudo-randomly presented stimuli. Two sequential blocks contained the speech sound stimuli (i.e.,/a/then/u/) and two sequential blocks contained the non-linguistic stimuli (i.e., 800 Hz then 400 Hz). For each category (vowels or tones), each stimulus was used as both the “flip” and the “flop” variant in the MMN paradigm. For half of the participants, the speech sound stimuli blocks were played first (TSC: *n* = 5; TD: *n* = 5); for the other half of participants, the non-linguistic blocks were played first. The inter-stimulus interval was 700 ms. The stimuli intensities were equalized to be 61 dB ± 3 dB when playing through two speakers positioned bilaterally in front of the participant. The stimuli intensities were measured using a sound meter from the distance that the participants sat from the speakers. Stimuli were presented via speakers instead of earphones due to the sensory sensitivities that are often present in individuals of this demographic. The stimuli were played using E-Prime experimental software (Psychology Software Tools Incorporated, Pittsburgh, Pennsylvania).

### Procedure

Participants sat in an electrically shielded and sound attenuated room. Participants passively listened to the stimuli while watching a silent video (Wall-E, Disney Pixar) on a computer monitor. An experimenter sat in the room to maintain participant engagement and ensure that participants continued to tolerate wearing the net. Breaks were provided as needed between the blocks of stimuli. The MMN procedure lasted for approximately 24 min. The stimuli were presented as a part of a battery of EEG paradigms. The entire battery was approximately 45 min long.

### EEG Recording

A continuous EEG recording was collected for each participant using a 128-channel Geodesic Sensor Net (Philips Electrical Geodesics Inc., Eugene, OR). The net size was determined by the child’s head circumference. EEG was recorded using Net Station Acquisition software (Philips Electrical Geodesics Inc., Eugene, OR) at a sampling rate of 1000 Hz and referenced online to the average reference. A Net Amps 300 amplifier was used to amplify the electrical signal.

### Data Processing

The data were processed offline using Net Station analysis software (Philips Electrical Geodesics Inc., Eugene, OR). A bandpass filter of 0.3–30 Hz was used. The continuous recording was segmented into 600-ms epochs, including 100-ms before the onset of the stimuli as a baseline. The mean voltage during the baseline period was used for baseline correction of each epoch.

Artifact detection was automated by the Net Station program. Channels within each segment were removed if the difference between the maximum and the minimum heights of the waveform exceeded 200 μV. Segments with more than 18 eliminated channels were excluded from further analysis. The standard stimulus segments that immediately follow a deviant stimulus were also removed. If the number of good segments varied by more than five segments per condition for a given participant, segments were randomly eliminated until all conditions were within five segments of each other. Participants with fewer than 10 good segments in any given condition were excluded from further analysis (*n* = 1). The average number of good segments in an included standard condition was 212, with a range of 70–252. The average number of good segments in an included deviant condition was 47, with a range from 22 to 54.

Bad channels within the accepted segments were replaced using a spherical spline interpolation. Average waveforms for each electrode referenced to the average reference were generated for each participant, and a final baseline correction was applied. Finally, the individual trials were averaged by condition for each participant.

For MVPA, the epoch data was processed as described above (i.e., bandpass filter, segmentation, baseline correction, artifact removal); however, we did not average across the trials for each electrode. Instead, after artifact removal we re-referenced and baseline corrected the single trial data and then exported the single trial data to MATLAB to run the MVPA.

### Analysis

#### MVPA

We utilized MVPA to explore the functional specificity of neural processing for differentiating standard speech sounds compared to standard non-speech stimuli. For each pairwise classification between the four standard stimuli, a linear classifier was trained in MATLAB on 3/4 of the trials for each participant. The other 1/4 of the trials were used to test the accuracy of classifier. We used a fourfold cross-validation with pseudo-averaging and 300 random permutations of the data (Isik et al., 2014; Grootswagers et al., 2017; Bayet et al., 2018). To prevent possible effects from the order of presentation, only trials from the second and third stimuli blocks (i.e., the two middle blocks) were used for each participant, thereby eliminating trials that are further separated by time (i.e., the first and last blocks of stimuli). The average number of included trials from a single stimuli block was 213, with a range of 70–252. Forty-eight of the electrodes were excluded from our analysis due to location on the outer rim of the electrode cap.

#### AEP Component Analysis

Waveforms were calculated from montages resulting from electrodes in the right frontal region. The right frontal region (electrodes 2, 3, 4, 10, 122, 123, 124) was selected as the region of interest (ROI) *a priori* to the analysis based on the implication of this region in the processing of vowel sounds and tones (Lepisto et al., 2005; Ceponiene et al., 2008; Britton et al., 2009). Further, because one of the strengths of MVPA is the identification of relevant electrode clusters during neural processing, we used the spatial resolution of the MVPA as evidence-based confirmation that the electrode clusters chosen *a priori* were indeed relevant to our paradigm. The average number of good trials included by group and condition are included in Table 2.

**TABLE 2:** Average number of good trials per condition, by group.

**Group**	**Vowels (standards)**	**Tones (standards)**	**Vowels (deviants)**	**Tones (deviants)**
TSC	370 SD = 108	377 SD = 114	83 SD = 17	85 SD = 19
Control	475 SD = 42	474 SD = 40	103 SD = 7	102 SD = 7

**FIGURE 2:**
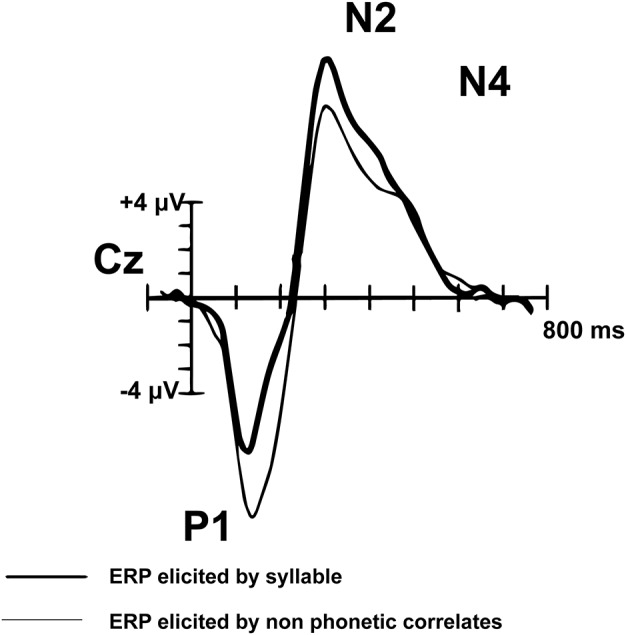
Pediatric (*n* = 14) syllable (bold) and non-speech ERP grand-average waveform from Cz (adapted from Ceponiene et al., 2005). The P1, N2, and N4 peaks are evident in the pediatric auditory waveform. In contrast to the matured adult AEP waveform, the N1 and P2 peaks are not observed in children.

Peak components of interest from the montage averaged waveform were selected based on their association with language processing in pediatric populations. Components of the AEP waveform mature through adolescence. The adult AEP waveform contains the following sequence of positive (P) and negative (N) inflections in response to speech and tones: P1, N1, P2, N2, N4. In contrast, children do not have noticeable N1 or P2 peaks in response to speech sounds or tones (Figure 2; Ceponiene et al., 2008). The P1, N2, and N4 components evident by early childhood (e.g., age 7) have been shown to reflect neural processes associated with basic auditory detection, recognition, and spectral changes in pitch and speech sound formants (Ceponiene et al., 2008). The N4 peak has been identified as particularly relevant to speech processing (Ceponiene et al., 2008). Adults and TD children demonstrate a reduced or absent N4 in response to tones, compared to a larger N4 response to speech sounds (Ceponiene et al., 2005). Data further suggest that diminished N2 and N4 peaks in response to consonant-vowel syllables are correlated with language impairment (Ceponiene et al., 2009). In light of the consistent P1, N2, and N4 in the developing pediatric AEP response, we explore these components of the auditory AEP as potential auditory biomarkers.

The waveform peaks were identified in each participant according to established pediatric time windows (Ceponiene et al., 2009): P1 (maximum positive peak between 70 and 190 ms), N2 (most negative peak between 270 and 390 ms), and N4 (most negative peak between 350 and 500 ms). The amplitudes and latencies of each peak were averaged across standard group category (i.e., responses to the standard/a/and/u/were averaged together, responses to the standard 800 Hz and 400 Hz were averaged together) by participant.

#### MMN

As described above, the right frontal region waveform response was also used when identifying the MMN. To confirm the presence of the MMN response, the minimum peak (between 100 and 300 ms) of the standard and deviant waveforms were compared for each condition.

To quantify the MMN, the difference waveform (response to the deviant minus response to the standard) was calculated for each stimulus between 100 and 300 ms (Lepisto et al., 2005). Difference waveforms were calculated on a per subject basis and the difference wave was used for analysis. The minimum negative peak between 100 and 300 ms was identified as the mismatch negativity amplitude. The presence of the MMN was analyzed at the population level; data was included from each participant regardless of whether an MMN was detected for an individual stimulus.

The literature suggests that the ASD diagnosis drives some EEG phenotypes in TSC, including the MMN response (Jeste and Nelson, 2009; Jeste et al., 2015). Acknowledging the limitations of our small sample size, we ran preliminary analyses to explore these possible trends in the MMN response of children with both TSC + ASD, compared to the MMN response of participants in the TD and TSC - ASD groups.

### Statistical Analysis

#### MVPA

For each participant group, the accuracy of the linear classifier was determined for decoding between (1) standard tones (800 Hz vs. 400 Hz); (2) standard speech sounds (/a/vs./u/); (3) standard tones vs. standard speech; and (4) all standard stimuli (e.g.,/a/vs./u/, 800 Hz vs. 400 Hz, etc.).

Accuracy vs. chance was analyzed for both of the time windows as defined above (corrected for multiple comparisons at the FDR level) and for a cluster-level correction over time points. In the latter case, statistical significance of the classification accuracy time-series against chance was established using permutation tests (right-tail test against the chance level of 50 or 0% as appropriate, 1000 permutations) with cluster-wise correction over time-points (cluster-defining threshold *p*-value = 0.05, α = 0.05) (Bayet et al., 2018; Dobs et al., 2018).

Additionally, we analyzed whether the classification accuracies (1) within and (2) between stimuli categories were above chance for each group. Average pairwise classification accuracies over two broad time windows of interest (early, 100–250 ms; late, 250–500 ms) were analyzed using Linear Mixed Effects (LME) Models to test for effects of group (TSC group/TD group) and classification type (within-domain classification, such as 400 Hz tone vs. 800 Hz tone, or cross-domain classification such as/a/vs. 800 Hz tone). A random intercept was used for each participant. Analyses of Variances (ANOVAs) were conducted to test the statistical significance of fixed effects, with follow up *t*-tests as appropriate.

All MVPA analyses were run in MATLAB (The MathWorks, Natick, MA).

#### AEP Component Analysis

To compare the amplitudes of each peak (P1, N2, N4), we completed a repeated-measures ANOVA for each peak with stimuli category (speech/tone) as within-factor and group (TSC group/TD group) as between-factor. To compare the latency of each peak (P1, N2, N4), we completed a repeated-measures ANOVA for each peak with stimuli category (speech/tone) as within-factor and group (TSC group/controls) as between factor. The AEP statistical analyses were performed with GraphPad Prism 7 for Mac OS X (GraphPad Software, Inc., La Jolla, CA). Significant main effects and interactions were followed up with unpaired *t*-tests between the groups. All analyses were corrected for multiple comparisons using Sidak’s test of multiple comparisons. All significance levels were set at α < 0.05. To compensate for the small sample size of our groups, *post hoc* Bayesian comparisons were conducted for the amplitude and latency of each AEP component using IBM SPSS Statistics version 25 (IBM Corp., Armonk, NY).

#### MMN Analysis

To confirm the presence of an MMN response in each group (TSC group/TD group), we used a *t*-test for both auditory conditions (speech/tone). Each *t*-test compared the minimum amplitude (between 100 and 300 ms) in response to the standard stimuli to that of the deviant stimuli for each group.

To compare the amplitudes of the MMN response between each group (TSC group/TD group), we used a *t*-test for both auditory conditions (vowels and tones). Each *t*-test compared the most negative amplitude of the difference waveform (deviant minus standard) between groups, for both the vowel and the tone conditions.

To explore possible trends in the MMN response driven by the ASD diagnosis, we used a one-way ANOVA to compare the three groups (TD group, TSC – ASD, TSC + ASD group) for both auditory conditions (vowels and tones). Significant effects were followed up with Dunnet’s Multiple Comparisons (TD group/TSC – ASD group, TD group/TSC + ASD group). All significance levels were set at α < 0.05. *Post hoc* Bayesian comparisons were also conducted using IBM SPSS Statistics version 25 to compensate for the small sample sizes.

The MMN statistical analyses were performed with GraphPad Prism 7 for Mac OS X (GraphPad Software, Inc., La Jolla, CA).

## Results

Please see Supplementary Table S1 for the results of all statistical analyses reported below, as well as results from *post hoc* Bayesian comparisons that further supported our findings.

### MVPA

There was a significant group x stimulus interaction [*F*(1,32) = 6.37, *p* = 0.0168], with more accurate decoding between stimulus class than within class in the TD group (*p* = 0.0214) but not the TSC group (*p* = 0.404) at an early time window (100–250 ms), indicating that there was significant difference in the neural response to tones vs. vowels in the TD group but not the TSC group.

**FIGURE 3:**
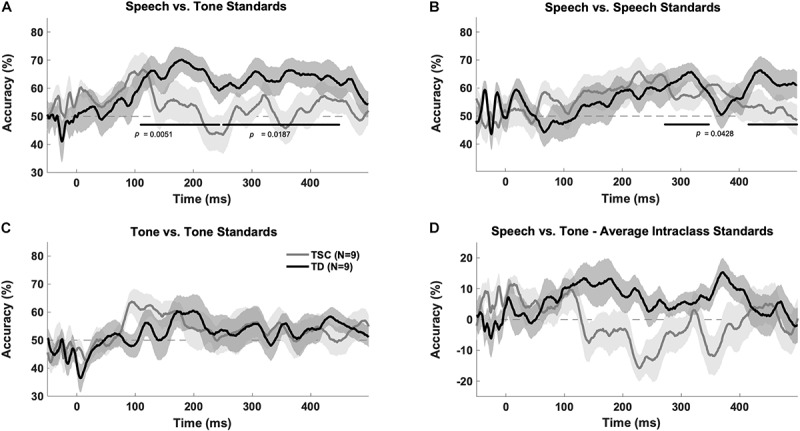
Multivariate pattern analysis accuracy between groups. Plots represent decoding accuracy between responses to speech sounds compared to tones (/a/ + /u/vs. 800 Hz + 400 Hz) **(A)**, the standard speech sounds (/a/vs./u/) **(B)**, tones (800 vs. 400) **(C)**, and between all stimuli (/a/vs./u/vs. 800 Hz vs. 400 Hz) **(D)** for the children with TSC and typically developing children. The bold lines on the figures indicate time points of above-chance decoding in the TD group, as determined by the cluster correction method. In contrast, the TSC data did not yield significant above-chance decoding at any time point. Levels of chance were set to 50% **(A–C)** and 0% **(D)** as appropriate.

The TD group had above chance decoding between speech vs. tones at early (100–250 ms; *p* = 0.005) and late (250–500 ms; *p* = 0.0187) time windows after FDR correction. A cluster analysis revealed above chance decoding between speech and tones from 110 to 449 ms for the TD group (*p* < 0.05). The TSC group did not have above chance decoding between these stimuli categories (early: *p* = 0.401; late: *p* = 0.416). A cluster analysis confirmed that decoding was not above chance in any time range for the TSC group (*p* > 0.05) (Figure 3A).

The MVPA revealed above chance decoding for the TD group within the speech category (/a/vs./u/) at a late time window (*p* = 0.0428) (as confirmed by the time-wise analysis finding of two significant clusters between 273–348 and 418–499 ms, *p* < 0.05), but not at an early time window (*p* = 0.176), after FDR correction (Figure 3B). The TSC group did not demonstrate above-chance decoding within the speech sound category at early (*p* = 0.133) or late (*p* = 0.133) time windows.

Unlike for speech, there was no above chance decoding between tones (400 Hz vs. 800 Hz) for the TD group at early (*p* = 0.176) or late (*p* = 0.176) time windows. Similarly, there was no above chance decoding between tones for the TSC group at early (*p* = 0.133) or late (*p* = 0.400) time windows (Figure 3C). Unexpectedly, there was above chance decoding for all stimuli within each class for the TD group at early time points (*p* = 0.0428), however, the cluster analysis at this time point was not statistically significant *(p* > 0.05). As expected, there was no above chance decoding for all stimuli within each class at late time windows (*p* = 0.416) for the TD group, or at early (*p* = 0.176) or late (*p* = 0.607) time windows for the TSC group (Figure 3D).

### AEP Waveform Analysis

**FIGURE 4:**
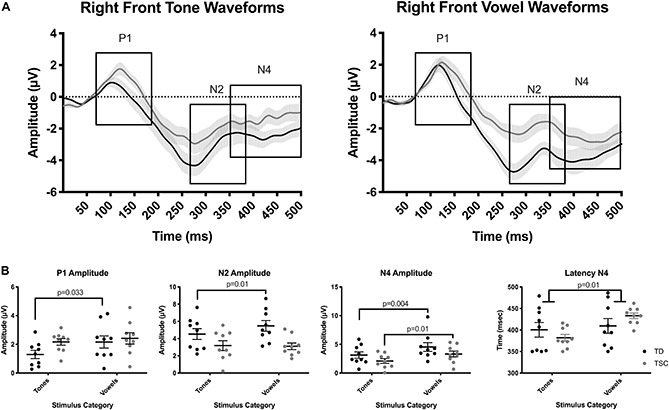
Auditory evoked potential response to tones and vowels between groups in the right front cluster. Plots represent AEP waveform response to tones and vowels **(A)**, and absolute value in microvolts of the amplitude of each waveform component of interest (P1, N2, N4) **(B)**. There was an enhanced P1 response to vowels compared to tones in the TD group, but not in the TSC group. The TD group had significantly greater N2 amplitude in response to vowels, but not tones, compared to the TSC group. There was a greater N4 amplitude in response to vowels than tones in both the TD and TSC groups. The TSC group had significantly longer latency of the N4 in response to speech compared to tones, which was not seen in the TD group. No group or stimulus differences were observed related to the latency of P1 or N2.

Average waveforms were generated for both the TD and the TSC groups in response to normal tones (both 400 Hz and 800 Hz together) and normal speech sounds (both/a/and/u/together), excluding the first normal stimuli after an oddball stimulus (Figure 4A). Averaged waveforms for each participant for both tone and speech can be seen in Supplementary Figure S1.

#### P1 Amplitude

We found a main effect of stimulus for the P1 response [*F*(1,16) = 6.34, *p* = 0.0229], with an enhanced P1 response to vowels compared to tones (*p* = 0.027) in the TD group, but not in the TSC group (*p* = 0.686). There was no effect of group [*F*(1,16) = 1.71, *p* = 0.209] or a group x stimulus interaction [*F*(1,16) = 1.947, *p* = 0.182] (Figure 4B). Bayesian comparisons supported these findings, as shown in Supplementary Table S1.

#### N2 Amplitude

The N2 amplitude differed significantly between groups [*F*(1,16) = 6.736, *p* < 0.02]. The TD group had significantly greater N2 amplitude in response to vowels (*p* = 0.0104), but not to tones (*p* = 0.187), compared to the TSC group (Figure 4B). There was no effect of stimulus [*F*(1,16) = 1.66, *p* = 0.236] or group x stimulus interaction [*F*(1,16) = 2.325, *p* = 0.147]. Bayesian comparisons supported these findings, as shown in Supplementary Table S1.

#### N4 Amplitude

In contrast to our hypothesis, N4 amplitude did not differ by group [*F*(1,16) = 2.179, *p* = 0.159]. There was a greater N4 amplitude in response to vowels than tones [*F*(1,16) = 24.35, *p* = 0.0001] in both the TD (*p* = 0.0035) and TSC groups (*p* = 0.0105; Figure 4B). There was no group x stimulus interaction [*F*(1,16) = 0.139, *p* = 0.715]. Bayesian comparisons supported these findings, as shown in Supplementary Table S1.

#### P1, N2, N4 Latency

There were no significant main effects of diagnosis (*p* = 0.264) or stimuli (*p* = 0.347) for latency of the P1 component. Similarly, there were no significant main effects of diagnosis (*p* = 0.190) or stimulus (*p* = 0.0802) for the N2 peak. For the N4 latency, Levene’s Test of Equal Variance determined that there was unequal variance for tones (*p* = 0.006) and vowels (*p* = 0.013). As shown by the individual data points plotted in Figure 4B, this difference in variance was due to the expectedly reduced, or absent, N4 response to tones but not vowels in the TD group. A non-parametric Related-Samples Wilcoxon Signed Rank Test revealed a significant effect of stimulus (*p* = 0.011), but not diagnosis (*p* = 0.845), for N4 latency (Figure 4B). Bayesian comparisons supported these findings, as shown in Supplementary Table S1.

### MMN

**FIGURE 5:**
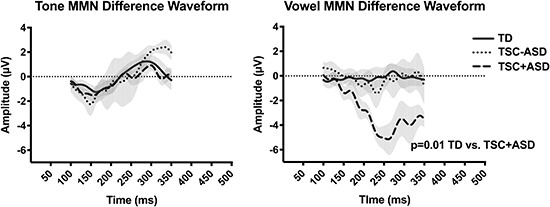
Mismatch negativity difference waveforms (deviant response – standard response) for controls and children with TSC by ASD diagnosis. MMN difference waveforms in microvolts in response to tones and vowels by groups at the right front cluster. No group differences are observed in the MMN response to tones or vowels between typically developing children and children with TSC. When exploring MMN response by ASD diagnosis, there was a significantly enhanced MMN response to vowels, but not tones, in the TSC + ASD group compared to the TD group.

Mismatch negativity waveform responses (indexing sound discrimination) were observed in the tone and vowel conditions for both groups. Unpaired *t*-tests revealed no significant difference between MMN amplitude between the TD and TSC groups in response to tones [*t*(16) = 1.117, *p* = 0.2806] or to vowels [*t*(16) = 1.499, *p* = 0.154] (Figure 5).

A one-way ANOVA exploring possible trends driven by the ASD diagnosis compared the three groups (TD, TSC – ASD, TSC + ASD) and revealed a significant difference between means of the three groups’ MMN response to vowels [*F*(2,15) = 4.39, *p* < 0.0174]. *Post hoc* comparisons using the Dunnet’s multiple comparisons test indicated that the mean of the TSC + ASD was significantly lower than the TD group (*p* = 0.0120), while the TSC – ASD group did not differ significantly in their MMN response from the TD group (*p* > 0.999) (Figure 5). There was no significant difference between the means of the three groups’ MMN response to tones [*F*(2,15) = 0.929, *p* = 0.416]. Bayesian comparisons supported these findings, as shown in Supplementary Table S1. Averaged difference waveforms for each participant for both tone and speech can be seen in Supplementary Figure S2.

## Discussion

Children with TSC have altered neural responses to vowels, but not tones, relative to TD children. MVPA suggests above chance decoding of speech vs. tones in the TD group but not in the TSC group. This is supported by AEP waveform analysis demonstrating an enhanced response to vowel sounds relative to tones in TD children but not in children with TSC. In the present study, TD children had an increased P1 amplitude to vowel sounds relative to tones and had a higher N2 amplitude to vowels than children with TSC. As expected, TD children had a reduced or absent N4 response to tones, compared to vowels. Children with TSC, though, did not demonstrate this absent N4 response to tones, and instead had a significantly longer latency for the N4 response than TD children. This could be due to less efficient processing, alternate processing pathways, or impaired conduction of neural signals in this population. These findings, coupled with the reduced connectivity in language related white matter tracts in this population (Lewis et al., 2013), suggest functional differences in basic speech detection for individuals with TSC.

A significant MMN response was elicited by deviant vowels and tones in both groups. We did not find a significant group difference in MMN amplitude or latency between the TD and TSC groups. In contrast to early AEP work in TSC (Seri et al., 1999), we found a preliminary trend toward an enhanced MMN response to vowels in children with TSC + ASD, as compared to the TD group and the TSC – ASD groups. This is consistent with other research that has shown an enhanced MMN in response to changes in speech pitch for children with ASD (Lepisto et al., 2005, 2008). Based on preliminary data, children with TSC + ASD, but not TSC – ASD, appear to have an enhanced attention to pitch change in speech sounds compared to TD children. This increased attention to unexpected speech sounds may contribute to downstream language processing difficulties.

Multivariate pattern analysis broadens traditional evoked potential analyses through the application of an unbiased approach to categorizing signals in both time and space. These results suggest that EEG responses from TD children show above-chance differentiation between stimulus category (speech vs. tones) in both early and late processing and within speech stimuli (/a/vs./u/) during late processing in the right frontal brain region. In contrast, EEG responses from children with TSC did not show this reliable differentiation between these stimuli categories during any time window. These outcomes potentially reflect fewer processing distinctions between speech and tones for individuals with TSC. It is also possible that children with TSC have increased heterogeneity in processing of speech and tones compared to the TD group, which may reduce the overall group-level accuracy of the linear classifier. Children with TSC contributed fewer valid trials to the analysis; although these trial numbers remain high, it is possible that the relatively lower number of valid trials could explain the lack of robust classification between auditory stimuli that was observed in this analysis. By adding MVPA as a complement to traditional methodology, we are taking full advantage of the temporal specificity that is provided from EEG in a manner that is less biased toward specific temporal windows and spatial regions in the data. It also allows for exploring cognitive variation in differentiating between stimuli, which may provide greater insight into cognition than traditional univariate analyses.

Despite the similar language abilities to the controls, as measured by a coarse language survey (Figure 1), we still detect basic processing differences between groups. Thus, the evoked responses to speech stimuli could represent a latent endophenotype related to language development more sensitive than overall verbal fluency. Despite the functional speech abilities of most of the participants, all individuals with TSC in our study receive speech-language therapy at school, suggesting the potential for the development of compensatory language processing strategies or language difficulties that were not fully detected by our brief parent survey. Validation of these measures as endophenotypes may provide a sensitive biomarker of language ability that could be used in clinical trials with language related outcome measures.

The apparent speech specific deficits in children with TSC are consistent with broader electrophysiological investigations into sensory processing in TSC. In the visual domain, research has demonstrated that infants with TSC do not have deficits in basic visual processing (i.e., as measured by the VEP in response to a changing checkerboard); however, adults with TSC + ASD do have deficits in socially relevant visual processing (i.e., faces) (Tye et al., 2015; Varcin et al., 2016). The current study explored basic and social processing in the auditory domain for children with TSC and found analogous outcomes: children with TSC do not have deficits in basic auditory processing (i.e., tones), however, they do have deficits in socially relevant auditory processing (i.e., speech sounds). Taken together, these outcomes suggest socially specific processing deficits in both the visual and auditory domains for individuals with TSC.

### Limitations

To validate sensory evoked responses for clinical applications, it will be necessary to broaden the study population. Due to inherent challenges associated with recruiting individuals with low-incidence genetic disorders, our sample size is relatively small and spans a wide chronological age range (4–14) and developmental co-morbidity. Further, our preliminary analyses of MMN as driven by a co-diagnosis of TSC + ASD include an even smaller subgroup of participants. Cross-site investigation are currently being undertaken to recruit larger numbers of this rare population, including participants across the developmental and chronological range proposed for clinical interventions in which similar measures are being collected. The age range included in this study spans a range in which there is a maturation of the AEP response, however, age related changes of the waveform are equally reflected in both the TSC and TD populations. Characterization of the speech evoked response across these domains will contextualize its utility as a biomarker.

## Conclusion

In this paper, we identify electrophysiological responses (P1, N2, N4) to vowel sounds, but not tones, that differentiate children with TSC from age and sex-matched controls. Processing differences suggest that children with TSC do not demonstrate the typical neural differentiation between speech compared to tones. A novel MVPA corroborates these traditional analyses with temporal specificity. The basic auditory processing deficits likely contribute to language difficulties seen in children with TSC and may serve as biomarkers of language impairment in the TSC population. Downstream effects of these basic auditory processing deficits (e.g., at the syllable, word, and sentence level) should be investigated further. Additionally, it is important to explore the responsiveness of these AEP components to behavioral and medical intervention to understand their clinical significance as biomarkers.

## Data Availability Statement

The data that supports the findings of these studies are available from the corresponding author upon reasonable request.

## Ethics Statement

The Boston Children’s Hospital Institutional Review Board approved this study. Informed written consent was provided by a parent or guardian of each participant. Participants provided written assent as appropriate.

## Author Contributions

AO’B contributed to the design of the study and analysis, contributed to the collection of the data, contributed to the data analysis and interpretation, and drafted the manuscript. LB contributed to the design of the MVPA analysis. KR contributed to the collection of the data. CN contributed to the collection of the data and revised the manuscript. MS contributed to the study design and revised the manuscript. MM conceived of the study, directed its design, coordination, analysis, and interpretation, and revised the manuscript. All authors read and approved the final version of the manuscript.

## Conflict of Interest

MM was previously employed by Pfizer but was not during the collection or analysis of these data and consults with BlackThorn Therapeutics. MS reports grant support from Novartis, Roche, Pfizer, Ipsen, LAM Therapeutics, and Quadrant Biosciences, and served on Scientific Advisory Boards for Sage, Roche, and Takeda. The remaining authors declare that the research was conducted in the absence of any commercial or financial relationships that could be construed as a potential conflict of interest. The authors declare that this study received funding from Pfizer Inc. The funder had the following involvement with the study: conceptual study design.

## Supplementary Material

FIGURE S1 Auditory evoked potential response to tones and vowels in the right front electrode cluster. Plots represents trial averaged waveforms from each participant for each stimulus type (tones and vowels). Response to both variants of the stimulus are included in the individual average.

FIGURE S2 Mismatch negativity difference waveforms (deviant response – standard response) for individual participants of each diagnostic group. Individual averaged waveforms were generated for both the deviant and the standard response and then subtracted for each participant to reveal the difference waveform.

TABLE S1 Detailed presentation of statistical analyses for each of the results depicted in the figures, including both traditional and Bayesian approaches.

AUDIO S1 800Hz.

AUDIO S2 400Hz.

AUDIO S3 /u/.

AUDIO S4 /a/.

## Abbreviations

AEP: auditory evoked potential
ASD: autism spectrum disorder
MVPA: multivariate pattern analysis
TSC: Tuberous Sclerosis Complex.
