# Supplementary figures and images for: Auditory Processing of Speech and Tones in Children With Tuberous Sclerosis Complex
Source: Front Integr Neurosci. 2020 Apr 9;14:14. doi: 10.3389/fnint.2020.00014 (PMC7161665; doi:10.3389/fnint.2020.00014)

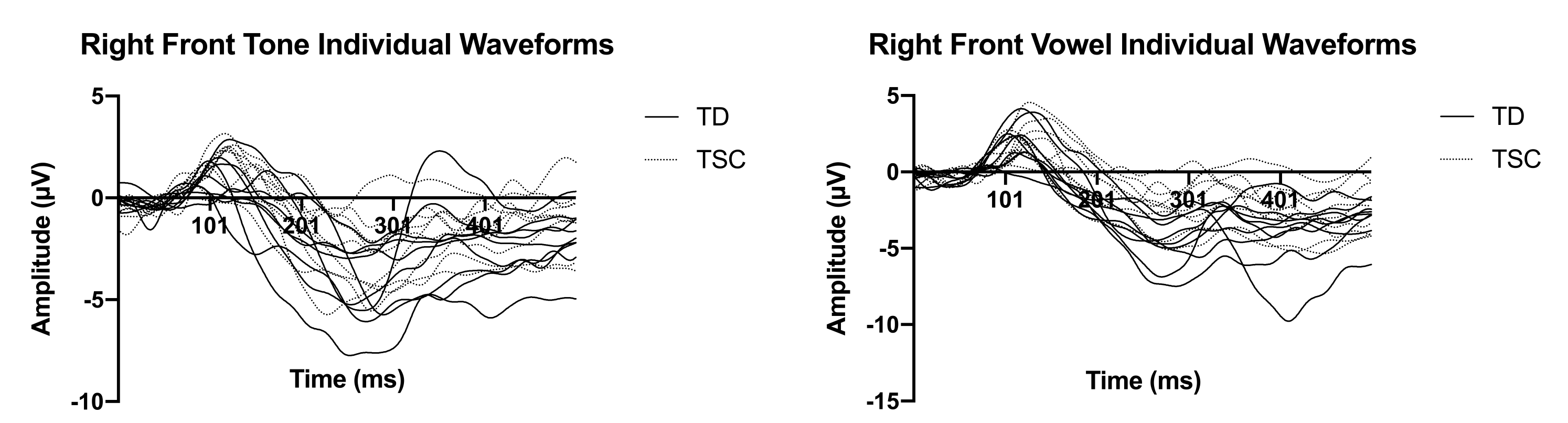

Supplement: FIGURE S1 — Auditory evoked potential response to tones and vowels in the right front electrode cluster. Plots represents trial averaged waveforms from each participant for each stimulus type (tones and vowels). Response to both variants of the stimulus are included in the individual average. [file Image_1.tif]

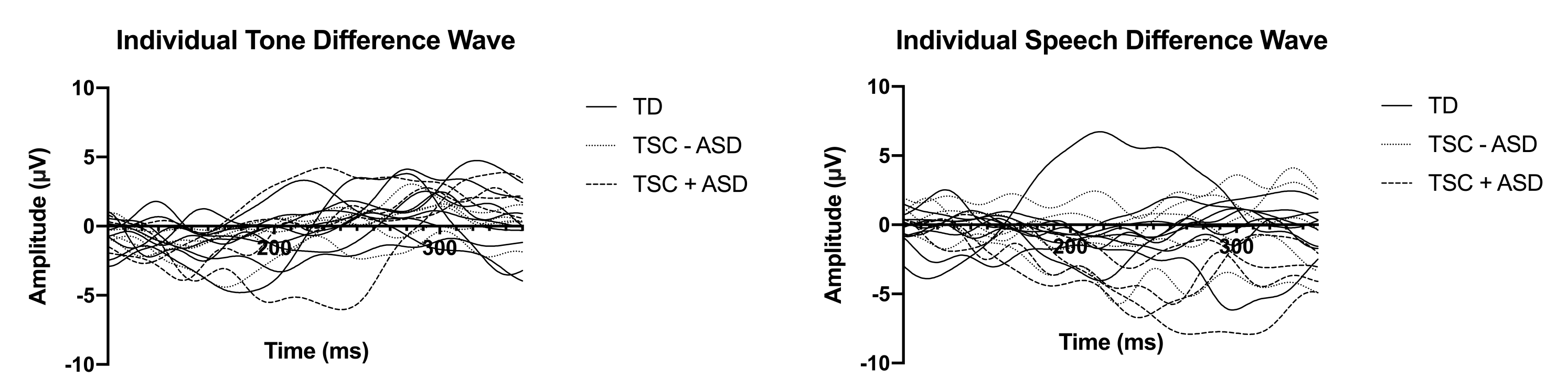

Supplement: FIGURE S2 — Mismatch negativity difference waveforms (deviant response – standard response) for individual participants of each diagnostic group. Individual averaged waveforms were generated for both the deviant and the standard response and then subtracted for each participant to reveal the difference waveform. [file Image_2.tif]
